# Chimpanzee identification and social network construction through an online citizen science platform

**DOI:** 10.1002/ece3.7128

**Published:** 2020-12-16

**Authors:** Maureen S. McCarthy, Colleen Stephens, Paula Dieguez, Liran Samuni, Marie‐Lyne Després‐Einspenner, Briana Harder, Anja Landsmann, Laura K. Lynn, Nuria Maldonado, Zuzana Ročkaiová, Jane Widness, Roman M. Wittig, Christophe Boesch, Hjalmar S. Kühl, Mimi Arandjelovic

**Affiliations:** ^1^ Max Planck Institute for Evolutionary Anthropology Leipzig Germany; ^2^ Department of Human Evolutionary Biology Harvard University Cambridge Massachusetts USA; ^3^ Taï Chimpanzee Project Centre Suisse de Recherches Scientifiques Abidjan Ivory Coast; ^4^ Éco‐corridors laurentiens Saint‐Jérôme Quebec Canada; ^5^ Zooniverse Citizen Scientist c/o Max Planck Institute for Evolutionary Anthropology Leipzig Germany; ^6^ Faculty of Medicine Institute for Drug Discovery Leipzig University Leipzig Germany; ^7^ iScapes Valencia Spain; ^8^ Department of Anthropology Yale University New Haven Connecticut USA; ^9^ German Centre for Integrative Biodiversity Research (iDiv) Halle‐Leipzig‐Jena Germany

**Keywords:** camera trap, chimpanzee, citizen science, *Pan troglodytes*, social network analysis

## Abstract

Citizen science has grown rapidly in popularity in recent years due to its potential to educate and engage the public while providing a means to address a myriad of scientific questions. However, the rise in popularity of citizen science has also been accompanied by concerns about the quality of data emerging from citizen science research projects. We assessed data quality in the online citizen scientist platform Chimp&See, which hosts camera trap videos of chimpanzees (*Pan troglodytes*) and other species across Equatorial Africa. In particular, we compared detection and identification of individual chimpanzees by citizen scientists with that of experts with years of experience studying those chimpanzees. We found that citizen scientists typically detected the same number of individual chimpanzees as experts, but assigned far fewer identifications (IDs) to those individuals. Those IDs assigned, however, were nearly always in agreement with the IDs provided by experts. We applied the data sets of citizen scientists and experts by constructing social networks from each. We found that both social networks were relatively robust and shared a similar structure, as well as having positively correlated individual network positions. Our findings demonstrate that, although citizen scientists produced a smaller data set based on fewer confirmed IDs, the data strongly reflect expert classifications and can be used for meaningful assessments of group structure and dynamics. This approach expands opportunities for social research and conservation monitoring in great apes and many other individually identifiable species.

## Graphical abstract

Citizen science has expanded rapidly in popularity, leading to a growing need to ensure data accuracy in citizen science research projects. We validated the accuracy of chimpanzee detection and individual identification data from the online citizen scientist platform Chimp&See, which hosts camera trap videos of chimpanzees and other species across Equatorial Africa. When comparing data from citizen scientists with those of experts who coded the same videos, we found a high level of agreement in detecting and identifying chimpanzees, although citizen scientists assigned fewer IDs overall. The resulting citizen scientist data could be used to construct a robust social network with a similar structure and network positions to that constructed from expert data coded from the same camera trap videos.

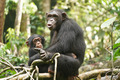

## 1 INTRODUCTION

The past decade has seen rapid growth in the breadth and popularity of citizen science programs (Gura, 2013; Silvertown, 2009). Citizen science offers researchers opportunities to amass, process, and analyze very large data sets efficiently, and in some cases to collect data that could not be collected otherwise (Frigerio et al., 2018; Nagy et al., 2012). In turn, citizen science provides opportunities to educate and engage the public on the scientific process (Frigerio et al., 2018). The development of novel technologies such as apps and online platforms has further permitted the growth and expansion of citizen science across a global scale (Frigerio et al., 2018).

Citizen science projects are particularly popular in the fields of biology and ecology (Kullenberg & Kasperowski, 2016), where they can provide opportunities to promote nature conservation and greater environmental awareness (Crall et al., 2011; Johnson et al., 2014). In these fields, such projects often center on the analysis of camera trap photographs or videos (e.g., Hsing et al., 2018; Swanson et al., 2015, 2016). Camera traps are a growing method of data collection as they allow for biological studies of ecosystems, as well as individual species and their behaviors (Caravaggi et al., 2017). For example, numerous recent studies in primatology have relied on camera traps (Pebsworth & LaFleur, 2014), which can serve as a tool for documenting novel behaviors (Boesch et al., 2017; Boyer Ontl & Pruetz, 2020; Kühl et al., 2016) and assessing group demographics and social dynamics (Galvis et al., 2014; McCarthy et al., 2018, 2019). The growing application of citizen science approaches to camera trap data poses exciting opportunities for processing large amounts of data in a variety of species.

However, enthusiasm for citizen science has been accompanied by concerns about the quality of data produced by these projects (Bonney et al., 2014; Kosmala et al., 2016). While some studies have demonstrated that citizen scientists produce data on par with expert data (Cox et al., 2012; Crall et al., 2011; Danielsen et al., 2014; Edgar & Stuart‐Smith, 2009; Nagy et al., 2012), others have found limitations in the accuracy of citizen science data (Foster‐Smith & Evans, 2003; Galloway et al., 2007; Gardiner et al., 2012; Gollan et al., 2012; Kremen et al., 2011; Moyer‐Horner et al., 2012). To overcome potential data quality concerns, citizen science projects should first evaluate data to ensure their accuracy. For example, citizen science can serve as a valuable tool for assessing species distribution and abundance (e.g., Foster‐Smith & Evans, 2003; Kremen et al., 2011; Moyer‐Horner et al., 2012; Swanson et al., 2015), but since such studies typically rely on accurate species identification from camera trap images, efforts should be taken to ensure data quality prior to making species assessments (e.g., Hsing et al., 2018; Swanson et al., 2015, 2016). In addition, such projects also commonly require accurate demographic assessments, for example, the number of individuals detected and the sex–age class of individuals (e.g., Delaney et al., 2007; Gardiner et al., 2012; Kelling et al., 2015), and here too, data quality can be evaluated to ensure accuracy prior to subsequent analyses.

Citizen scientists may also participate in identifying individual animals. Accurate individual identification (ID) is often a prerequisite for estimating abundance and studying social dynamics (Karanth et al., 2006; VanderWaal et al., 2009), although observer experience can affect the reliability of identification skills (Van Horn et al., 2014; Patton & Jones, 2008). Therefore, it is necessary to assess the reliability of citizen scientist observations prior to relying on these data for subsequent analyses, particularly since identification errors can lead to analytical biases such as the overestimation of species abundance (Johansson et al., 2020; Stevick et al., 2001). Although machine learning has also advanced considerably in recent years, allowing the potential for automated detection of individuals from recorded images (Schofield et al., 2019), its implementation still poses challenges (Green et al., 2020). Simultaneously, the benefits of citizen science are numerous, suggesting that machine learning and citizen science can work as integrated, complementary approaches to collecting high‐quality data on individual animals (Green et al., 2020).

We assessed individual IDs of chimpanzees (*Pan troglodytes*) made by citizen scientists using the online citizen science platform Chimp&See (www.chimpandsee.org). Chimp&See allows citizen scientists to classify camera trap videos recorded at research sites across Equatorial Africa as part of the Pan African Programme: The Cultured Chimpanzee (PanAf; http://panafrican.eva.mpg.de), a broad, cross‐sectional project that aims to elucidate drivers of behavioral diversity in chimpanzees. To assess chimpanzee classifications, we compared detections and individual identifications made by citizen scientists using a consensus‐based approach with those of scientists with extensive direct knowledge and experience studying the chimpanzees in the videos. In particular, we compared the following: (a) the number of individual chimpanzees detected in camera trap video clips, (b) the frequency with which individual IDs could be assigned, and (c) the agreement level for individual ID assignments made by citizen scientists and experts. Further, we examined whether ID disagreements typically followed meaningful patterns, for example, by citizen scientists assigning the same age class and sex as experts but differing in their ID assignment.

We applied the citizen scientist ID data for social network construction in these chimpanzees and compared it with a social network constructed from the expert ID assignments. Social network analysis offers a powerful means to study social group structure and dynamics and can be applied for numerous research and conservation applications (Farine & Whitehead, 2015). Additionally, recent research showed that robust chimpanzee social networks can be constructed using camera trap data from experts (McCarthy et al., 2019). Therefore, we assessed whether the individual IDs obtained from citizen scientist data could be applied to construct a robust social network with a similar structure to that constructed from expert data. We expected the citizen scientist data to contain more missing observations than data from experts with years of experience studying these chimpanzees, but previous research has shown that an individual's position within its social network can be determined accurately even with some unidentifiable individuals (Silk et al., 2015). Therefore, we also compared individual network positions in social networks constructed from both expert and citizen scientist data sets.

## 2 MATERIALS AND METHODS

### 2.1 Data collection

#### 2.1.1 Camera trap data collection

Camera traps (Bushnell Trophy Cam**™**;Model #119576C) were installed from July 2014 through March 2015 in the territory of habituated western chimpanzees (*Pan troglodytes verus*) of the Taї Chimpanzee Project in Taї National Park, Côte d’Ivoire (5°08’N to 6°07’N, and 6°47’W to 7°25’W; Després‐Einspenner et al., 2017; Wittig & Boesch, 2019). Chimpanzees live in territorial social groups, typically referred to as “communities,” that display fission–fusion dynamics, with group members associating in “parties” that vary in size, composition, and duration (Goodall, 1986). Previous research on this chimpanzee community has shown that camera trap videos coded by experts provide a valuable means to assess variation in chimpanzee party size and social dynamics (McCarthy et al., 2018, 2019). A mean of 64 (maximum = 83) camera traps was installed throughout the 40‐km^2^ home range of one chimpanzee community, termed the “East Group,” as previously described in detail (Després‐Einspenner et al., 2017; McCarthy et al., 2018). The East Group comprised between 32 and 36 total individuals at any given time during the study period. Camera traps were installed in either systematic (*N* = 23) or targeted (*N* = 107 total) locations. Systematic locations were within 30 m of intersection points of a 1 x 1 km grid overlaid onto the chimpanzees’ home range, while targeted locations were those areas frequently used by chimpanzees, for example, natural bridges, feeding sites, and tool use sites. Targeted cameras were moved to another location in the same grid cell if they failed to record chimpanzees for one month. Camera traps were motion‐triggered and recorded for 60s at a time, with a minimum 1‐s retrigger interval between videos.

#### 2.1.2 Expert identification of chimpanzees from camera trap videos

For research regarding population density, demography, and social dynamics in these chimpanzees (Després‐Einspenner et al., 2017; McCarthy et al., 2018, 2019), two observers, each with several years of experience studying and identifying the Taї chimpanzees, watched camera trap videos of East Group chimpanzees (*N* = 594 total videos) and identified individuals and their associated age class and sex. For a subset of 25% of these videos, both observers independently identified weaned chimpanzees (*N* = 24), and their interobserver agreement was assessed using the Cohen's kappa coefficient, which calculates agreement while taking into account the potential for chance concordance (Cohen, 1960). Their coefficient was 0.91 (McCarthy et al., 2019), which is typically considered in the range of nearly perfect agreement (Viera and Garrett, 2005). In the current study, as in previous research using this data set (McCarthy et al., 2019), weaned chimpanzees were defined as those older than 5 years of age, when weaning generally occurs and individuals begin to travel more independently (Goodall, 1986). Weaned individuals were of particular focus, because weaning generally marks the age at which chimpanzees are both: 1) reliably identifiable and 2) actively choose the individuals with whom they affiliate in parties, thereby justifying their inclusion in social network analyses. Any disagreements in chimpanzee identification (*N* = 25 identifications) were resolved with subsequent viewing, and a consensus agreement was made (*N* = 14), or in cases of poor video quality or incomplete visibility of the individual, the identity of the chimpanzee was coded as “unidentified” (*N* = 11).

#### 2.1.3 Chimp&See online citizen science platform

Chimp&See (https://chimpandsee.org), launched in 2015, is an online citizen science platform developed and hosted by The Zooniverse (https://www.zooniverse.org) to allow citizen scientists to view and annotate data from PanAf camera trap videos recorded across 39 temporary research sites in Equatorial Africa. Citizen scientists are recruited primarily via The Zooniverse, social media, word of mouth, and citizen scientist project portal websites (e.g., SciStarter: https://scistarter.org). The online platform allows anyone to participate irrespective of experience level and provides a brief tutorial at the beginning of classification, along with species identification guides and tips for individual identification.

Citizen scientists classify all species observed in 15‐s clips, as well as annotating the number of animals detected and their basic behaviors (e.g., traveling, feeding). These clips are derived from 60‐s camera trap videos, quartered to avoid attentional fatigue, and presented in random order. When chimpanzees are detected, citizen scientists are prompted to annotate the general age class (youth/adult) and sex of each individual in the clip before they can complete the classification.

**Figure 1:**
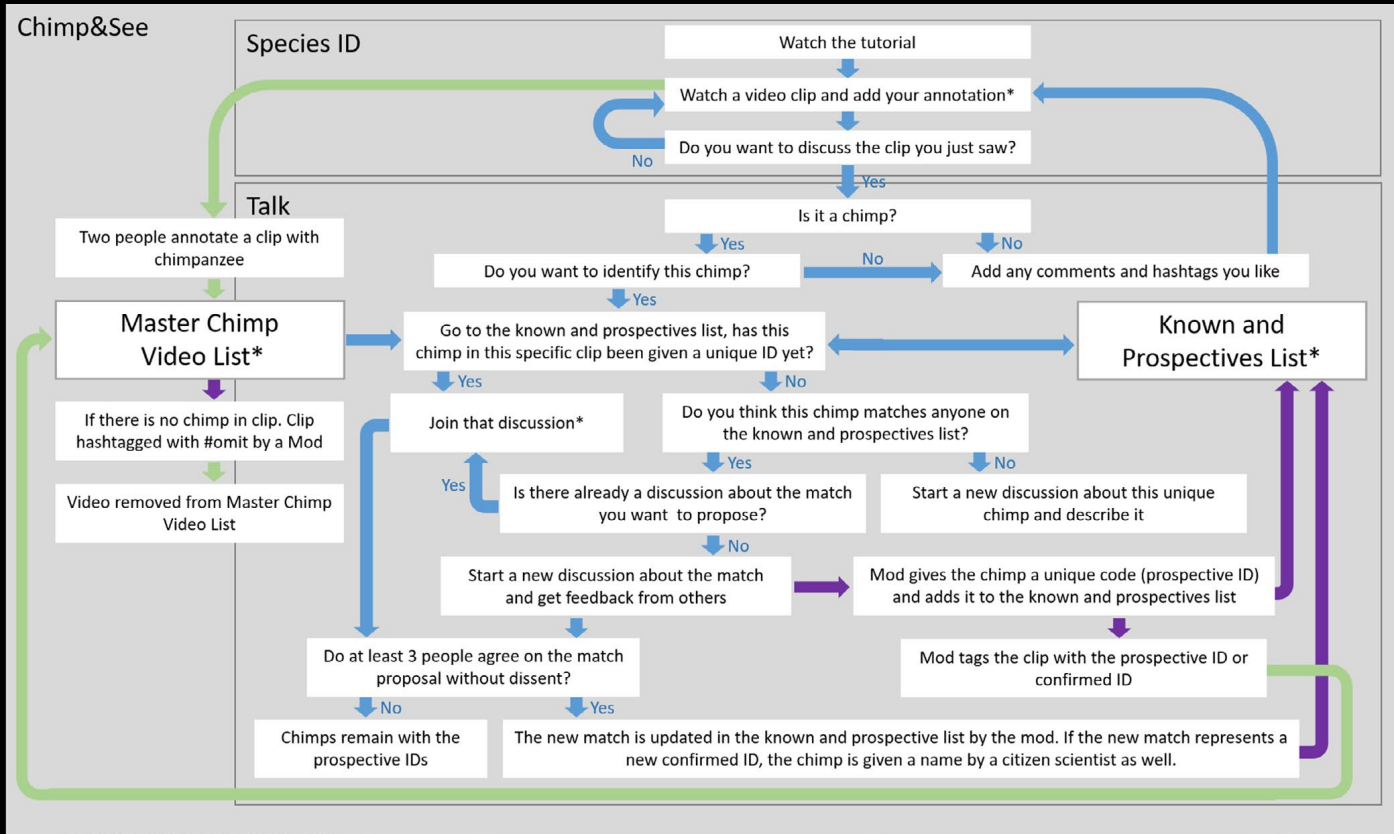
Workflow for chimpanzee identification on the Chimp&See platform. Citizen scientists can begin identifying video clips with chimpanzees in the species ID workflow or at any of the steps marked with an asterisk (*). Blue arrows are steps done by citizen scientists (including moderators). Green arrows are steps that are done automatically via custom Python scripts. Purple arrows are steps undertaken by the moderators only. The “Master Chimp Video List” is located off‐platform in an open access Google sheet

Following these independent chimpanzee classifications, citizen scientists are provided with the option to participate in an online discussion forum for chimpanzee identification moderated by a scientific moderator, a member of the PanAf science team with experience in both using the online platform and identifying chimpanzees. When a new chimpanzee is identified in a video clip, citizen scientists use the discussion forum to post videos and still images of the individual and describe individually identifiable features such as body size, hair color, facial markings, and scars. The scientific moderator then assigns a prospective ID (a temporary shorthand name that includes the site code, sex/age class, and a numeric identifier, e.g., “AVFem01”). When a minimum of three people agree that a chimpanzee sharing these features appears in at least two temporally and spatially independent video clips (i.e., nonconsecutive clips or clips from different camera locations), the individual is given a confirmed ID (a name chosen by one of the citizen scientists, e.g., “Penny”). By ensuring that consensus was established conservatively through agreement of at least three citizen scientists, we aimed to minimize ambiguous identifications and ensure more reliable ID data over a single person's assessment or pair in agreement. Photographs and descriptions of each confirmed individual are assembled in a list, curated by the scientific moderator, that is updated continuously as new prospective and confirmed individuals are identified. Subsequent identifications of each individual are confirmed by a moderator only after at least three people agree to a matching proposal. If any citizen scientist dissents from a matching proposal, the ID is left as unconfirmed unless a consensus is later reached (i.e., the dissenter later agrees to the proposed match in light of additional evidence). Prospective and confirmed identifications are indicated with name hashtags, which moderators enter into textboxes that caption each video clip. In addition to chimpanzee ID hashtags, citizen scientists use the textboxes to record additional data such as counts of the number of chimpanzees detected in each clip, notable behaviors, species detected, and other notes. Figure 1 presents a workflow diagram for classifications on Chimp&See.

Classification and hashtag results are saved in a digital database, MongoDB datastores, and are associated with each video clip identifier. After retrieving up‐to‐date classification and hashtag information using the Zooniverse API (application programming interface), a Python script uses these files to determine which clips are marked as containing chimpanzees, either by having at least two independent chimpanzee classifications (as a single chimpanzee classification could be erroneous) or by a “#chimp” hashtag caption. These clips are then assembled into a Google sheet, along with the video clip identifier, timestamp information from the video, link to the clip, associated hashtags, and other relevant notes to facilitate matching. Citizen scientist moderators check through the list and can use the moderator‐specific hashtag “#omit” to exclude videos in which chimpanzees are not detected so that they are removed from the list automatically, as can occur due to occasional errors (*N* = 198 in this study), such as if a citizen scientist accidentally clicks to classify a verifiably blank clip as containing chimpanzees. Since videos from any given site comprise thousands of clips of various species, this streamlined process for identifying and curating chimpanzee clips allows citizen scientists to efficiently subset these clips for further data collection while also filtering the inclusion of erroneous non‐chimpanzee clips.

### 2.2 Data analysis

#### 2.2.1 Detection of chimpanzees in camera trap videos

First, we assessed the number of chimpanzees detected in each video by citizen scientists and experts. We limited the comparison to 305 1‐min videos, a set comprising those videos: (a) in which all four 15‐s clips were viewed by citizen scientists and the corresponding 1‐min video was viewed by experts, and (b) in which citizen scientists identified at least one of four 15‐s clips as containing a chimpanzee and each of the other 15‐s clips comprising the 1‐min video as either also containing a chimpanzee or unanimously blank in three independent classifications. These criteria ensured that both groups viewed each 1‐min video in full and correctly identified it as containing at least one chimpanzee, thereby allowing valid comparisons of detection performance between the two groups. Next, since camera trap videos are cut into four distinct 15‐s clips for viewing on Chimp&See, we combined the four associated clips from the same minute to create comparable data from both experts and citizen scientists. In order to ensure accurate and comparable chimpanzee count data, we carefully linked citizen scientist data on individual chimpanzees across the four clips of each minute by using citizen scientist descriptions (i.e., of chimpanzee physical appearance, travel order, and position), individual ID hashtags, and chimpanzee count hashtags, thereby creating a tally of the total number of chimpanzees detected in each 1‐min video by experts and citizen scientists, respectively. Using these data, we compared the number of chimpanzees detected by experts and citizen scientists.

#### 2.2.2 Assignment rate and agreement for individual chimpanzee IDs

Next, we assessed how frequently citizen scientists and experts assigned individual IDs to chimpanzees detected in videos. From the individual chimpanzees detected by both groups, we determined the proportion to which IDs were assigned in these 305 1‐min chimpanzee videos.

Specifically, we determined the number and percentage of chimpanzee detections to which: (a) an expert ID and (b) a citizen scientist ID (either prospective or confirmed) were assigned, as well as the total number of distinct IDs assigned by each group and the mean number of instances in which each ID was assigned.

Then, we assessed agreement of individual ID assignments made by citizen scientists and experts. In particular, we aimed to assess agreement between citizen scientist‐confirmed IDs and expert IDs. To do so, we first standardized all IDs across data sets so that distinct names could be matched to determine agreement across the two data sets (e.g., the chimpanzee known as “Willy” by experts was given the ID “Caruso” by citizen scientists). One of the two experts who had previously assigned the expert chimpanzee IDs viewed a curated list containing all prospective and confirmed Chimp&See IDs assigned by citizen scientists. For each ID, a series of still images and Chimp&See video clips was provided. In each of these clips and images, there was consensus agreement on chimpanzee ID by the citizen scientists. The expert reviewed each citizen scientist ID and recorded a corresponding expert ID. In one case, the expert noted that a citizen scientist‐confirmed ID was associated with two distinct expert IDs. The expert identified all photographs and videos for that confirmed ID as matching one particular expert ID except one video clip, which she attributed to a different expert ID. In all other cases, each citizen scientist ID could be matched unambiguously to a particular expert ID.

After standardizing Chimp&See IDs for comparison with expert IDs, we assessed how frequently IDs matched. When videos contained multiple chimpanzees, we used annotations on chimpanzee behavior, position, and travel order, recorded ad libitum in both data sets, to assess whether IDs were in agreement for each chimpanzee detected. We then determined the percentage agreement between citizen scientist‐confirmed IDs and expert‐assigned IDs. When IDs assigned to a single chimpanzee were not in agreement, we assessed whether mismatching IDs followed a particular pattern, for example by occurring within the same age class–sex category as the expert ID.

#### 2.2.3 Comparing chimpanzee social networks

We used the confirmed IDs provided by the citizen scientists and the expert IDs to construct social networks based on association data for each data set. Videos were classified into events, such that all videos recorded at the same camera location within 15 min of each other were grouped into a single event (mean = 1.8 chimpanzee videos per event in this data set) (McCarthy et al., 2018, 2019). All individuals recorded in a given event were considered to be in association and members of the same party, and we used a simple ratio index to construct weighted adjacency matrices for dyadic associations among those individuals recorded together in events (Cairns & Schwager, 1987). The citizen scientist and expert networks comprised all weaned individuals identified in both data sets (*N* = 20 chimpanzees), and association data were based on all events containing at least one of these individuals (*N* = 171 and 271 events for the citizen scientist and expert networks, respectively).

To compare social network structure between the expert and citizen scientist data sets, we examined “network communities,” subgroups within the social network structure denoting individuals who tend to associate more with one another than others in the social group (Croft et al., 2008). We compared the number and composition of network communities in each social network using the packages “igraph” (Csardi & Nepusz, 2006) and “asnipe” (Farine, 2013) in R version 3.5.2 (R Core Team, 2018). In addition, we also used a multiple regression quadratic assignment procedure (MRQAP) with double semi‐partialling and 1,000 permutations to compare the similarity of the network structures (Dekker et al., 2007) using the R package “asnipe” (Farine, 2013). The MRQAP is a form of the Mantel test that relies on a Monte Carlo permutation method to determine significance while controlling for autocorrelations in matrix regressions (Dekker et al., 2007).

We examined social network robustness in the citizen scientist data by determining whether the number of observations obtained from the citizen scientist data was sufficient to find stable social network structure. We did so by constructing 32 estimated networks from the citizen scientist network. Each estimated network was constructed from a subset of the full data, beginning at a sample size of 15 events with each subsequent estimated network adding 5 observations up to 170 events, approximating the complete data set of 171 events (Davis et al., 2018; McCarthy et al., 2019). To assess network robustness, we used rank correlations to compare network structure between each estimated network and the complete network (Davis et al., 2018; McCarthy et al., 2019). We calculated the mean and 95% confidence intervals by bootstrapping the data used to construct the estimated networks 1,000 times (Davis et al., 2018; McCarthy et al., 2019).

We compared individual network positions in each of the social networks with two common measures, eigenvector centrality and strength, using the R package “igraph” (Csardi & Nepusz, 2006). Both are individual measures that can be used to assess an individual's relative position and gregariousness in their social network through the strength of their direct and indirect associations (Farine & Whitehead, 2015). We used the Spearman rank correlations to assess the similarity in individual network positions in both data sets (Siegel & Castellan, 1988).

## 3 RESULTS

Chimp&See data collection for the Taї East Group site occurred from 13 June 2016 to 26 January 2018. During this data collection period, 1,744 registered citizen scientists made 658,208 classifications (including blank clips, as well as chimpanzees and all other species detected in camera trap videos). Eighteen citizen scientists provided substantive contributions to individual chimpanzee identifications.

### 3.1 Detection of chimpanzees in camera trap videos

In total, experts detected 1,068 chimpanzees in 305 videos, while citizen scientists detected 1,035 chimpanzees in these same 305 videos. Experts and citizen scientists agreed on the number of chimpanzees in 87% of videos (*N* = 266/305 videos). In cases of disagreement, citizen scientists tended to detect fewer (69% of disagreements, *N* = 27, range = 1–8 fewer chimpanzees) rather than more (31%, *N* = 12, range = 1–3 more chimpanzees) chimpanzees compared with experts. Mean disagreement in the number of individuals detected was 1.6.

### 3.2 Assignment rate and agreement for individual chimpanzee IDs

Of the chimpanzees detected in these 305 videos, experts assigned IDs to 907 of 1,068 detected chimpanzees (85%), while citizen scientists assigned confirmed IDs to 474 of 1,035 detected chimpanzees (46%).

Overall, experts identified 36 unique chimpanzees in the videos they viewed (*N* = 594), and these included all individuals known to be present during the study period except for one, an infant born near the end of camera trap data collection but never captured on video (McCarthy et al., 2018). These 36 individuals represent a 3% underestimate in total chimpanzee abundance during the study period. Each expert ID was assigned a mean of 47.2 times (range = 1–121).

In contrast, citizen scientists assigned confirmed IDs to 29 individual chimpanzees across all videos they viewed (*N* = 574), which represents a 22% underestimate of the total number of chimpanzees known to be present during the study period (*N* = 37, *N* = 32 – 36 at any given time). Each citizen scientist ID was assigned 24 times on average (range 2–77). Citizen scientists also assigned 40 prospective IDs to individuals that could be described with unique features but could not be determined with consensus as either matching any confirmed ID, nor as a unique individual. Of these 40 prospective IDs, an expert coder identified 18 (45%) as corresponding to a chimpanzee with a confirmed ID, that is, an individual identified elsewhere in the citizen scientist data set but not confirmable in that particular video. Nine prospective IDs corresponded to chimpanzees that were either unidentifiable by experts due to poor video quality or were not members of the East Group. The other prospective IDs (*N* = 13) represented unique individuals that were not assigned a confirmed ID in the data set, with each unique individual being represented by between one and six prospective IDs. Only two chimpanzees that appeared in the camera trap videos were never assigned either a prospective or confirmed ID by the citizen scientists. These individuals were a mother–infant pair who died early in the study period and were only identified once in the camera trap videos by the expert coders.

For chimpanzee detections in which both citizen scientists and experts assigned a confirmed ID (*N* = 471), their IDs agreed in 99% of cases (*N* = 468) and disagreed < 1% of the time (*N* = 3). These disagreements comprised the following: (a) experts identifying a particular mother–female infant pair, while citizen scientists identified a mother–male infant pair; and (b) experts identifying a particular adolescent female, while citizen scientists identified a different adolescent female.

### 3.3 Comparing chimpanzee social networks

**Figure 2:**
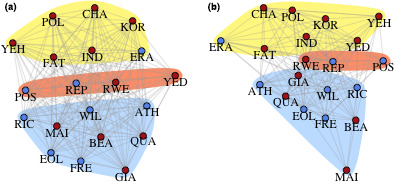
Chimpanzee social networks constructed from (a) citizen scientist and (b) expert data. Shaded regions indicate network community membership using the leading eigenvector method (Newman, 2006). Circles indicate individual chimpanzees (nodes) with blue and red indicating male and female chimpanzees, respectively. Lines (edges) connecting the nodes indicate dyadic associations among individuals

Both social networks contained three network communities, and 95% of the individual chimpanzees were found in the same network community in both networks; only one individual was in a different network community in the expert network than in the citizen scientist network (Figure 2). The social network structure based on the citizen scientist data significantly predicted the social network structure based on the expert data (Adj. *R* ^2^ = .63; *p* < .001).

**Figure 3:**
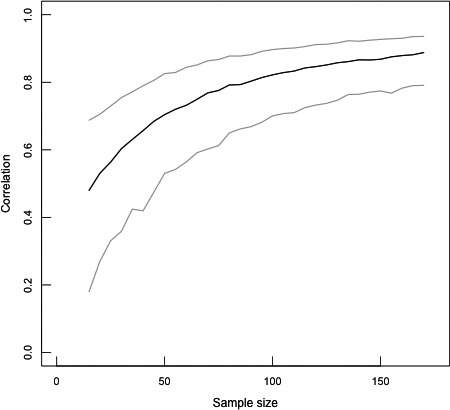
Mean (black line) and 95% confidence intervals (gray lines) for rank correlations of network structure at increasing sample sizes in the citizen scientist social network

**Figure 4:**
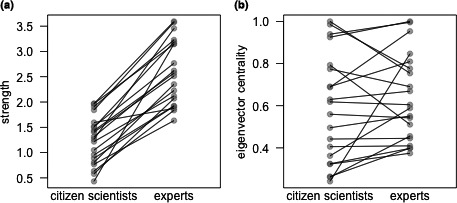
Individual measures for strength (a) and eigenvector centrality (b) among the weaned chimpanzees identified in both expert and citizen scientist networks (*N* = 20). Dots indicate the measures for each individual chimpanzee, and the lines connect the same individuals’ positions in the citizen scientist and expert networks, respectively

The association network constructed from the citizen scientist data was based on a smaller sample of events than the network constructed from expert data (*N* = 171 and 271 events for the citizen scientist and expert data, respectively) due to fewer IDs being assigned in the citizen scientist data. Nonetheless, the social network constructed from the citizen scientist data displayed relative stability at the complete sample size of 171 events, whereby a larger data set would lead to only a modest increase in rank correlations and reductions in the size of the confidence interval (Figure 3). This is similar to the 100–150 observations found to lead to social network stability when using either the expert data from camera trap observations or when using data derived from direct field observations of these chimpanzees for social network construction (McCarthy et al., 2019). Individual measures of network position were positively correlated in the expert and citizen scientist networks (eigenvector centrality: *r* = .62; strength: *r* = .59; Figure 4).

## 4 DISCUSSION

Citizen science projects have increased sharply in number and participation in recent years. The accessibility, educational opportunities, and entertainment value of online citizen science platforms make them particularly appealing as tools for scientific outreach and data collection. Nonetheless, it is critical to assess data quality from such projects prior to applying these data for basic research and applied conservation purposes. Using the online citizen science platform Chimp&See, we found that citizen scientists typically detected the same number of chimpanzees in camera trap videos as experts did. Disagreements in the number of chimpanzees detected were typically small (mean = 1.6) and more often underestimates, with a few outlier underestimates being due most likely to rare and temporary technical problems of the platform or other sources of stochastic error whereby citizen scientists classified 15‐s chimpanzee video clips as blank.

Of those chimpanzees detected, citizen scientists confirmed individual identifications far less frequently than experts. This is perhaps not surprising, given that the experts had years of experience studying and identifying these chimpanzees via direct observation. With the investment of more time and additional citizen scientists, it may have been possible to assign a small number of additional IDs, but considering the practical limitations of video quality and the time required to achieve consensus agreement in a small number of additional cases, these extensive efforts would have yielded relatively little and would have been unlikely to change the results we found. Nonetheless, confirmed IDs provided by citizen scientists nearly always agreed with those provided by experts. This is promising, since accurate but incomplete ID assignments can still produce meaningful results (Silk et al., 2015), and can lead to far less egregious errors than larger data sets containing identification errors (Davis et al., 2018). In our study, extremely rare cases of disagreement involved different IDs for chimpanzees of the same‐age class, and for mature individuals, the same sex as well.

We used these ID assignments to construct social networks from both expert and citizen scientist data. The social network structure based on the citizen scientist data significantly predicted the network structure based on the expert data. These networks also contained the same number of network communities, each with nearly identical composition, and the individuals’ relative positions in their social networks were positively correlated using both approaches. The network based on citizen scientist data achieved relative stability at approximately 150 observations, such that additional increases in sample size led to small reductions in the size of bootstrapped confidence intervals but only minor increases in the correlation with complete network structure. This is similar to what has been observed previously for social networks constructed for this chimpanzee community using expert‐coded camera trap data and direct observational data (McCarthy et al., 2019). Critically, this indicates that despite having fewer confirmed IDs in the citizen scientist data, robust social network construction is still possible.

It is important to note that our findings do not take into account the varying ID skill levels among experts and citizen scientists, respectively. Experts who have studied great apes, but not these particular individuals, may have potentially identified individuals less accurately than the experts in this study, who have had years of experience studying these particular chimpanzees and are therefore highly knowledgeable and skilled at identifying them accurately. However, these expert coders were previously found to have a level of ID agreement on par with that among video coders who identified unknown chimpanzees with moderate knowledge of chimpanzee behavior, although the latter group required much more time to identify individuals (McCarthy et al., 2019). Citizen scientists also vary in their level of prior experience, skill, and efficiency, and independent ID skills may vary among individuals in ways that could not be detected using our consensus‐ and discussion‐based approach for chimpanzee identification. Future research should further examine how individual identification varies based on factors such as experience level, and how the efficiency of this process may be maximized while ensuring data accuracy.

As citizen science methods are refined, machine learning algorithms continue to advance automated detection, including for individual great apes (Kühl & Burghardt, 2013; Schofield et al., 2019). However, this technology requires many training videos per individual and is not yet optimized for discerning large numbers of individuals captured in camera trap videos, which vary widely in resolution and lighting conditions across often densely forested great ape habitats. Citizen scientist data have the advantage of not relying on such technological constraints. In addition, citizen science beneficially engages the public in research, which may also serve to educate and inspire an increased appreciation of nature while fostering a sense of community among like‐minded individuals working toward a shared set of goals (Brossard et al., 2005; Gura, 2013; Toomey & Domroese, 2013). The Chimp&See online community comprises over 17,500 registered citizen scientists to date, and has fostered broad and inclusive collaborations and scientific engagement in countries around the world.

Our results suggest promising applications for integrating camera trap data with citizen science. Nevertheless, progressive enhancements in camera trap technology are likely to be accompanied by further improvements in methods for increasing the accuracy and efficiency of camera trap data collection and analysis. Camera trap videos are being recorded and uploaded with higher quality and increasing efficiency, with the potential to view videos in real time as they are recorded. Video analysis by citizen scientists may be aided by hybrid approaches involving automated machine learning for species detection prior to citizen scientist identification of individuals and behaviors, making applications for research and conservation more streamlined and efficient (Green et al., 2020; Willi et al., 2019). Citizen science data accuracy can also be enhanced in various ways, for example by providing pretraining to citizen scientists (Nagy et al., 2012), or by taking advantage of experience citizen scientists already have (Silvertown et al., 2015) or that they acquire through their involvement in a citizen science project (Kelling et al., 2015; Swanson et al., 2016). This can also be achieved through methods to maximize data accuracy through discussion‐based consensus‐building approaches for identifying individuals (Cox et al., 2012; He & Wiggins, 2015) or by aggregating individual assessments using a “wisdom of the crowd” approach (Swanson et al., 2016). The consensus‐based approach we used through Chimp&See led to highly reliable ID assignments, further supporting this method. In addition, accuracy may be enhanced through the use of statistical techniques to overcome systematic data issues (Bird et al., 2014).

These methods for individual assignment can be applied to citizen science projects targeting numerous species with identifiable features beyond primates, for example, cheetahs (Brassine & Parker, 2015), tigers (Karanth et al., 2006), Andean bears (Van Horn et al., 2014), and cetaceans (Würsig & Jefferson, 1990). Our results also illustrate that social network approaches hold great potential as an application of individual ID data from camera traps. Social network analysis is a highly useful means by which to assess social structure and dynamics (Johnson et al., 2017), as well as the transmission of information (Aplin et al., 2012), diseases (Silk et al., 2017), and behaviors (Hobaiter et al., 2014) across a wide range of species. These data can shed light on the evolution and ecology of organisms, as well as their responses to anthropogenic threats (Goldenberg et al., 2016), thereby guiding conservation measures to aid in their continued survival. During the Anthropocene, as more species are threatened with extinction worldwide (Ceballos et al., 2015), high‐quality data from citizen scientists are likely to be viewed as an increasingly valuable asset for research and conservation.

## CONFLICT OF INTEREST

None declared.

## AUTHOR CONTRIBUTION

Maureen S McCarthy: Conceptualization (supporting); Data curation (equal); Formal analysis (lead); Methodology (supporting); Visualization (lead); Writing‐original draft (lead); Writing‐review & editing (lead). Colleen Stephens: Conceptualization (supporting); Data curation (equal); Formal analysis (equal); Investigation (supporting); Software (supporting); Writing‐review & editing (supporting). Paula Dieguez: Data curation (lead); Investigation (supporting); Methodology (supporting); Project administration (supporting); Supervision (equal); Writing‐review & editing (supporting). Liran Samuni: Data curation (supporting); Investigation (supporting); Methodology (supporting); Writing‐review & editing (supporting). Marie‐Lyne Després‐Einspenner: Data curation (equal); Funding acquisition (equal); Investigation (supporting); Methodology (supporting); Writing‐review & editing (supporting). Briana Harder: Data curation (supporting); Software (supporting); Writing‐review & editing (supporting). Anja Landsmann: Data curation (supporting); Software (supporting); Writing‐review & editing (supporting). Laura K Lynn: Data curation (supporting); Software (supporting); Writing‐review & editing (supporting). Nuria Maldonado: Data curation (supporting); Investigation (supporting); Methodology (supporting); Writing‐review & editing (supporting). Zuzana Ročkaiova: Data curation (supporting); Software (supporting); Writing‐review & editing (supporting). Jane Widness: Data curation (supporting); Software (supporting); Writing‐review & editing (supporting). Roman M Wittig: Funding acquisition (equal); Methodology (supporting); Project administration (equal); Resources (supporting); Supervision (equal); Writing‐review & editing (supporting). Christophe Boesch: Conceptualization (supporting); Funding acquisition (equal); Methodology (supporting); Project administration (equal); Supervision (equal); Writing‐review & editing (supporting). Hjalmar Kuehl: Conceptualization (supporting); Funding acquisition (equal); Methodology (supporting); Project administration (equal); Supervision (equal); Writing‐review & editing (supporting). Mimi Arandjelovic: Conceptualization (equal); Data curation (supporting); Formal analysis (supporting); Funding acquisition (equal); Methodology (supporting); Project administration (lead); Supervision (lead); Writing‐review & editing (supporting).

## Data Availability

Chimpanzee ID matching data are available in the Dryad repository (https://doi.org/10.5061/dryad.xd2547dfx).
